# Supplementary material for: The G-Protein-Coupled Estrogen Receptor (GPER) Regulates Trimethylation of Histone H3 at Lysine 4 and Represses Migration and Proliferation of Ovarian Cancer Cells In Vitro
Source: Cells. 2021 Mar 11;10(3):619. doi: 10.3390/cells10030619 (PMC8001910; doi:10.3390/cells10030619)
Supplement: Supplementary file 1 [file cells-10-00619-s001.pdf]

Supplementary Materials:

1. The Effect of G36 on Caov3 and Caov4 Cells

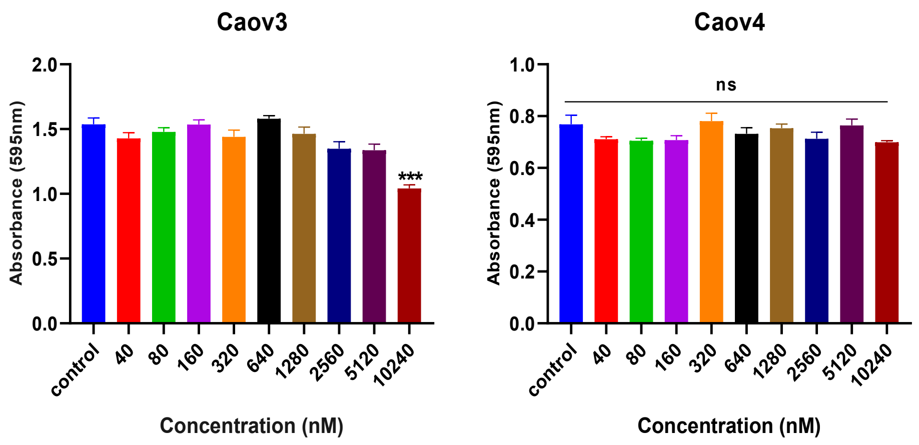

Figure S1. G36 had no effect on Caov3 and Caov4 cells. Both ovarian cancer cell lines were incu-bated with an increasing concentration of G36 for 24 h and the cell growth was evaluated by MTT assay. The result was presented as effective absorbance at 595 nm. Each experiment was repeated six times (n = 6) and the results expressed as the means ± SEM. Statistical analysis were performed by one-way ANOVA test (ns>0.05, *** $p < 0.001$ vs. control group).

2. GPER Expression after G1 or G15 Treatment for 24 h in Caov3 and Caov4 Cell Lines

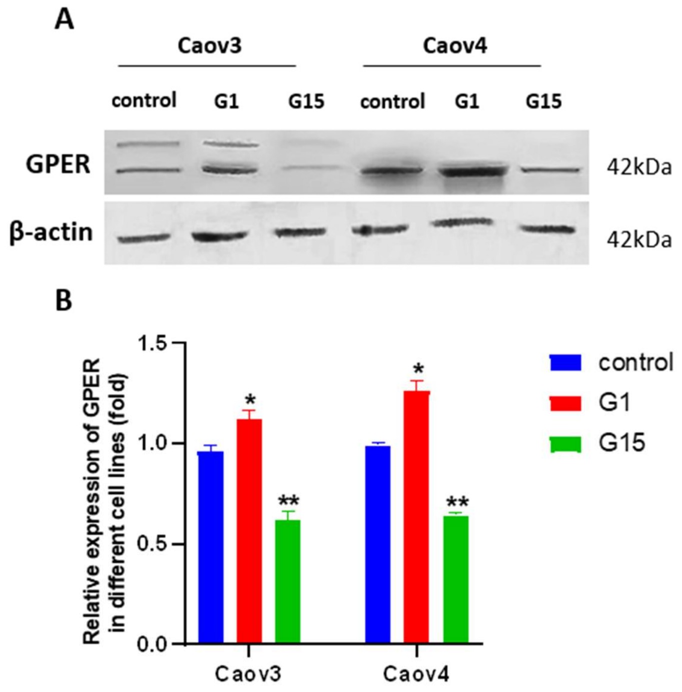

Figure S2. Caov3 and Caov4 cells were treated with 1 μM G1 or 1 μM G15 for 24 h. The levels of GPER protein were detected by western blot analysis. (**A**) Representative example of GPER protein expression in Caov3 and Caov4 cells after G1 or G15 treatment. (**B**) Histogram illustrates the ratio of GPER in Caov3

and Caov4 cell lines. * p < 0.05, ** p < 0.01 compared with control. β-actin was used as a loading control. Each experiment was repeated at least three times. The results are shown as the means ± SEM.

3. M30 Cyto-Death Assay

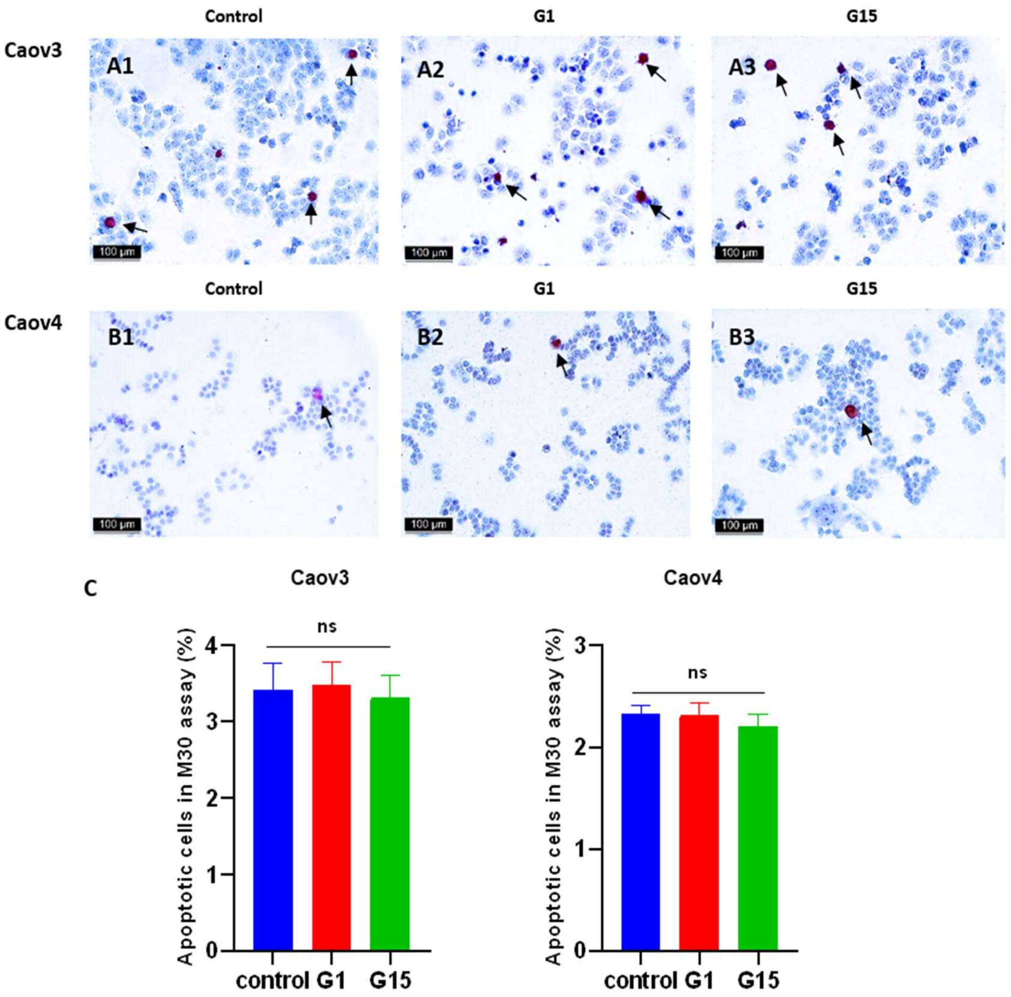

Figure S3. The detection of apoptotic cells using the M30 antibody after treatment with G1 or G15 for 24 h. The upper panel shows Caov3 cells treated with G1 and G15, respectively: (**A1**) control; (**A2**) G1; (**A3**) G15. The middle panel shows Caov4 cells treated with G1 and G15 respectively: (**B1**) control; (**B2**) G1; (**B3**) G15. The apoptotic cells stand out due to red staining (arrows) and negative cells show blue. (**C**) Results of M30 assay are summarized (n = 10). M30-positive cells are expressed as the number of positive cells ×100%/total number of cells analyzed. Values are expressed as the mean ± SEM. Scale bar = 100 μm.

4. *H3K4me3 Immunocytochemistry Staining of Caov3 and Caov4 Cells after G1 or G15 Treatment for 24 h*

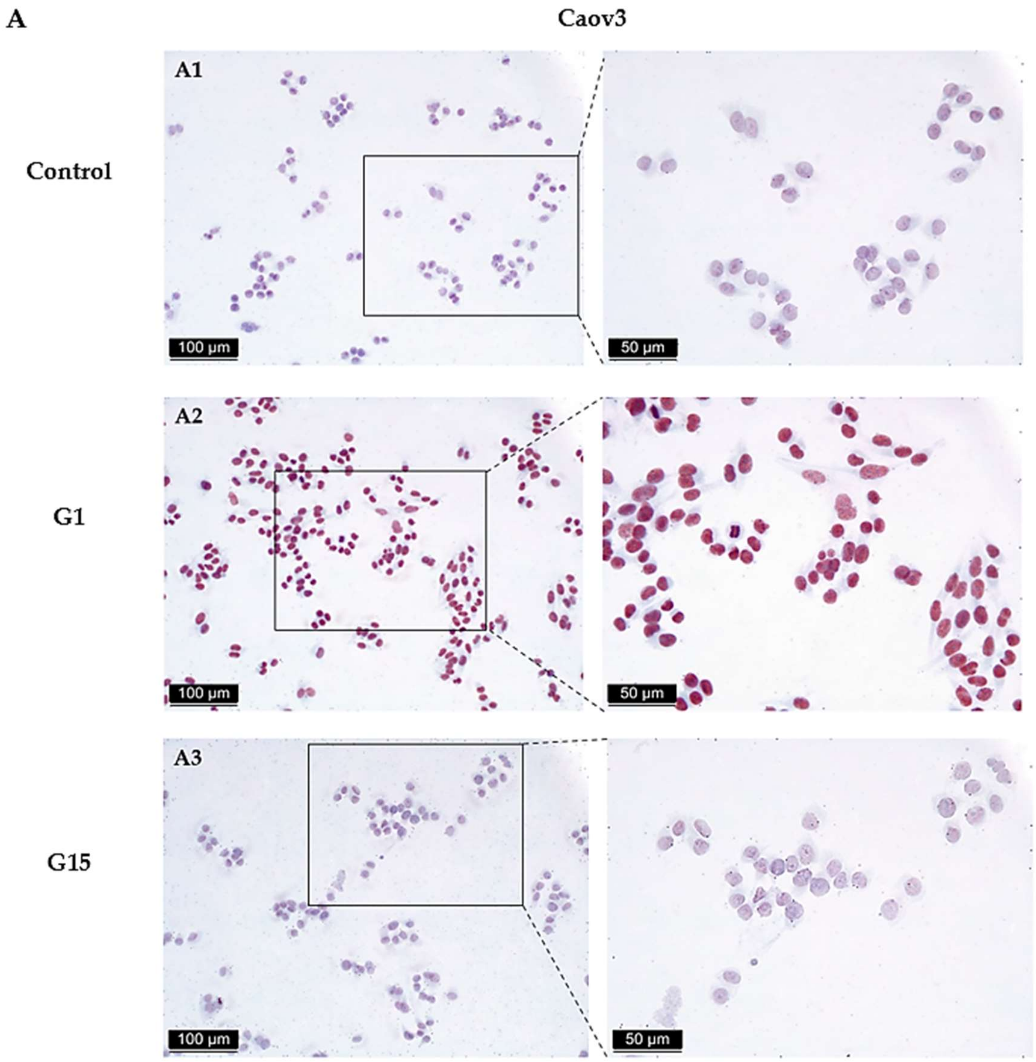

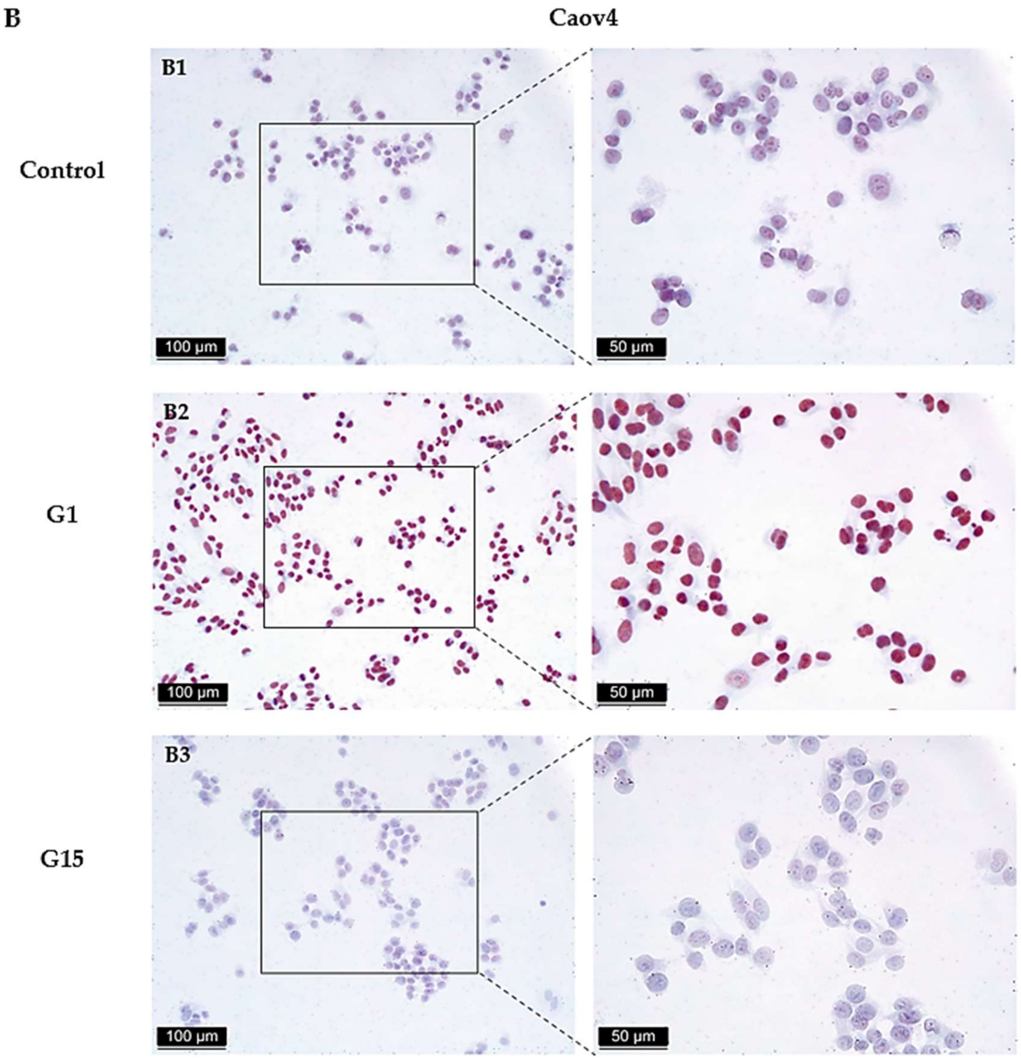

Figure S4. Representative pictures of H3K4me3 immunocytochemistry staining of Caov3 (**A**)and Caov4 (**B**) cells compared to the control (**A1**, **B1**), 1 μM G1 (**A2**, **B2**) and 1 μM G15 (**A3**, **B3**) for 24 h. Red staining is positive and blue staining is negative. Left panel: scale bar = 100 μm; right panel: scale bar = 50 μm.

5. Positive and Negative Controls for GPER and H3K4me3 in Placental Tissue

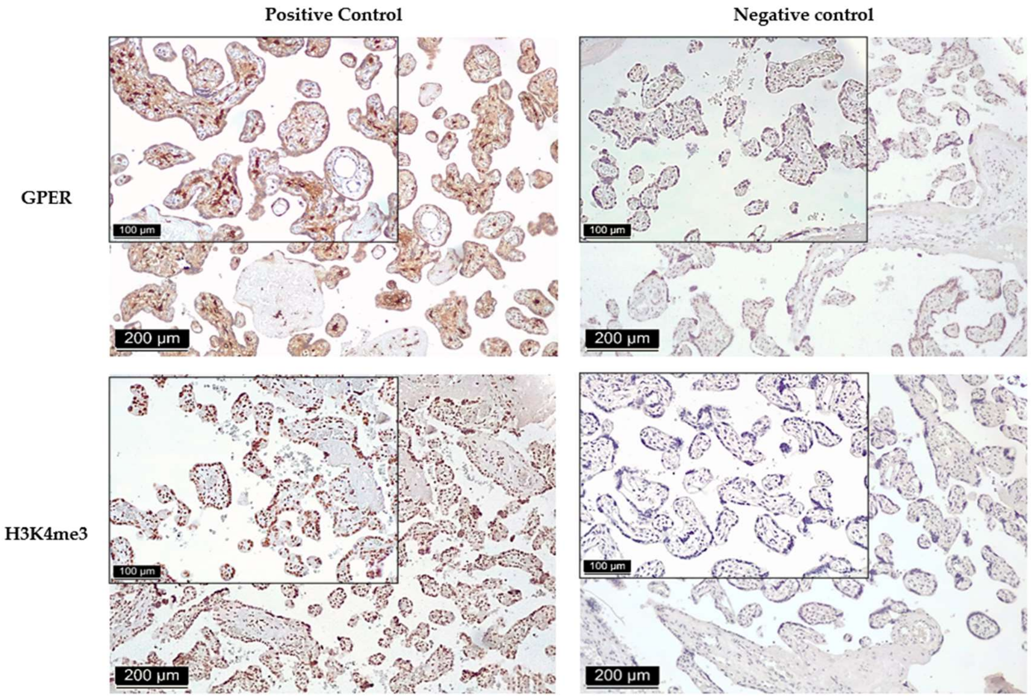

Figure S5. Positive and negative controls of the GPER and H3K4me3 stainings, respectively. We used the term placental villous tissue for positive and negative controls (scale bar = 200 µm, small pictures 100 µm).
